# Metatronics-inspired high-selectivity metasurface filter

**DOI:** 10.1515/nanoph-2024-0123

**Published:** 2024-04-26

**Authors:** Qihao Lv, Xu Qin, Mingzhe Hu, Peihang Li, Yongjian Zhang, Yue Li

**Affiliations:** Department of Electronic Engineering, 12442 Tsinghua University, Beijing 100084, China; Beijing National Research Center for Information Science and Technology, Beijing 100084, China

**Keywords:** metasurface filter, high-selectivity, metatronics, dispersion synthesis

## Abstract

Metatronic circuits extend the concept of subwavelength-scaled lumped circuitry from electronics to optics and photonics, providing a distinctive design paradigm for versatile optical nanocircuits. Here, based on the design of optical nanocircuits using metatronics concept, we introduce a general approach for dispersion synthesis with metasurface to achieve high-selectivity filtering response. We theoretically and numerically demonstrate how to achieve basic circuit lumped elements in metatronics by tailoring the dispersion of metasurface at the frequency of interest. Then, following the Butterworth filter design method, the meticulously designed metasurface, acting as lumped elements, are properly stacked to achieve a near-rectangular filtering response. Compared to the conventional designs, the proposed approach can simultaneously combine high selectivity with the theoretically widest out-of-band rejection in a considerably simple and time-efficient manner of circuit assembly, similar to electronic circuits, without extensive numerical simulations and complex structures. This dispersion synthesis approach provides exciting possibilities for high-performance metasurface design and future integrated circuits and chips.

## 1 Introduction

Filtering devices are indispensable in various applications like wireless communications [1], biomedical detectors [2] and image sensors [3], for the removal of unwanted signals. Within these applications, the high-selectivity filtering devices are required to have high rectangular coefficients (steep transition bands) to improve spectrum utilization efficiency and avoid wasting spectrum resources. Beyond high rectangular coefficients, high-selectivity filtering devices are also required to be with broadband out-of-band rejection. This property is vital for various signal-processing systems to eliminate the interference caused by spurious signals from adjacent bands in the current crowded spectrum resources.

In the past decades, enormous efforts have been devoted to achieving high-selectivity filtering devices, although the aspect of out-of-band rejection bandwidth is often ignored [4], [5], [6], [7], [8], [9], [10], [11], [12], [13], [14], [15], [16], [17], [18], [19], [20], [21], [22], [23], [24], [25]. In the microwave and THz regimes, an effective method for improving rectangular coefficients is to introduce transmission zeros by employing cross-coupling paths through substrate integrated waveguide cavities [4], [5], [6], [7] and microstrip multimode resonators [8], [9], [10], [11], [12] in frequency selective structures (FSSs). However, this method comes with the cost of compressing the out-of-band bandwidth, because the high-order passband, which should be located at twice of the frequency of the operating band, is red-shifted due to the additional transmission modes caused by the coupling of multiple resonant modes, making it difficult to achieve broadband out-of-band rejection for both the lower and upper sides simultaneously. Moreover, due to the lack of accurate analytic solution between the cross-coupling modes and the structural dimensions, the final design still requires extensive numerical simulations to optimize numerous dimension parameters of the complex structures. In the infrared and optical regimes, high-selectivity performance is usually implemented by exploiting high-order multistage resonators, such as Bragg gratings [13], [14], [15], [16], [17], microrings [18], [19], [20], [21], [22] and photonic crystals [23], [24], [25]. However, such high-selectivity response is achieved at the expense of both in-band and out-of-band bandwidths. In addition, since the abovementioned devices are inherently based on the interference phenomena, their dimensions in the wave propagating path are inevitably much larger than the operating wavelength.

Recently, the development of metatronics provides us with an advanced approach for designing filtering devices through dispersion synthesis [26], [27], [28], [29], [30], [31], [32], [33]. In the paradigm of metatronics, basic lumped elements, such as capacitors, inductors, and their series and parallel configurations, are usually physically implemented using structures and materials with different dispersions. Over the past few years, metasurfaces and metamaterials have been extensively used to enact complex electromagnetic functionalities, attributed to their unique capacity to tailor dispersion by adjusting the types and dimensions of the employed resonators [34], [35], [36], [37], [38], [39], [40]. This advantage enables the meticulously designed metasurface to emulate the traditional lumped elements in electronic circuits, thereby manipulating the flux of displacement currents to achieve various frequency dispersions. It is worth mentioning that unlike earlier work to achieve FSSs based on the equivalent circuit model, the employed metasurfaces in the framework of metatronics essentially act as actual circuits to control the flow of displacement currents. The benefit of this approach is that metatronics are inherently simple, unlike conventional FSS designs where equivalent impedances are defined by solving the many-body scattering problem through extensive numerical simulations, metatronic lumped elements are characterized by structures with known impedance expressions, physically connected to control the flow of displacement currents. In this way, under the guidance of metatronics, complex functional circuits can be realized by strategically embedding meticulously designed metasurface as lumped circuit elements in proper circuit locations without relying on enormous numerical simulations, surpassing the capabilities of conventional FSSs and metasurfaces.

In this paper, we introduce a general approach for dispersion synthesis with metasurfaces to yield high-selectivity combined with broadband out-of-band rejection performance according to the concepts of metatronics. Theoretical formulae are derived to provide a comprehensive map between resonant parameters of double-resonant metasurface model and basic metatronic lumped elements, including inductors, capacitors, series and parallel *LC* pairs. Then, a slot-type periodic metasurface is designed to facilitate parallel *LC* pairs necessary for high-selectivity filtering circuit. Following the Butterworth (maximally flat) filters design method, multi-layered parallel *LC* pairs inspired by metasurfaces with predetermined dimensions are stacked to realize high-selectivity combined with theoretically widest out-of-band rejection property. Theoretical results based on this metatronic circuit approach are verified by full-wave simulations and experiments. The proposed approach can be extended to any frequency dispersion synthesis, thus offering exciting possibilities for high-performance metasurface design.

## 2 Results

**Figure 1:**
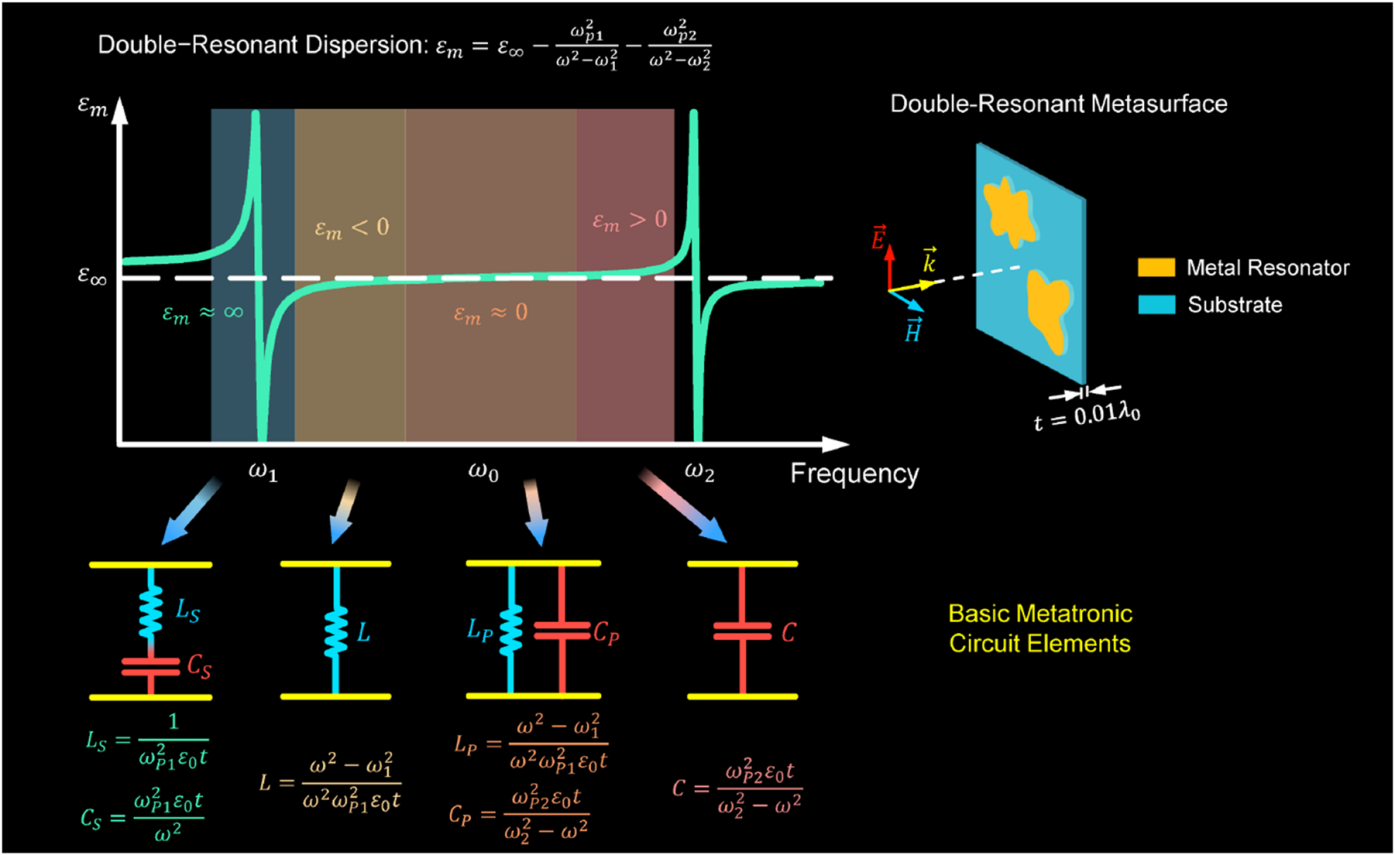
Comprehensive map of double-resonant metasurfaces and basic metatronics circuit elements. The double-resonant metasurfaces act as inductors, capacitors, series and parallel *LC* pair at different dispersion region of permittivity.

Figure 1 illustrates a comprehensive map between the resonant parameters of double-resonant metasurfaces and basic metatronic circuit elements, including inductors, capacitors, series and parallel *LC* pairs. The responses of double-resonant metasurfaces in free-space can be modularized as a lumped element with the admittance derived as follows:

(1) $$Y_{m}=-j\omega \left(\varepsilon_{m}-\mu_{m}\right)\varepsilon_{0}t,$$

where *ɛ*_*m*_ and *μ*_*m*_ denote the relative permittivity and permeability of the metasurfaces, respectively, *t* is the thickness, *ɛ*_0_ is the permittivity in vacuum, and *ω* is the operating frequency. Detailed theoretical derivation of equation (1) is demonstrated in Supplementary Note 1. This equation reveals that the metatronic circuits can be tailored by adjusting the permittivity of nonmagnetic metasurfaces (*μ*_*m*_ = 1). Here, the thickness of the metasurfaces is set to 0.01*λ*_0_ to better satisfy the requirement of the subwavelength-scale thickness in equation (1) and match the concept of “lumpedness” in metatronics. Considering that the relationship between metatronic circuit lumped elements and their corresponding double-resonant metasurfaces is built through real permittivity and is independent of the imaginary permittivity, in the following analysis, for simplicity and without the loss of generality, we assume the permittivity of the metasurface to be real.

In a double-resonant metasurface, whose relative permittivity follows the dispersion

$$\varepsilon_{m}=\varepsilon_{\infty }-\frac{\omega_{p1}^{2}}{\omega^{2}-\omega_{1}^{2}}-\frac{\omega_{p2}^{2}}{\omega^{2}-\omega_{2}^{2}}.$$

Here, *ɛ*_∞_ = 1 is the relative permittivity at infinite frequency, *ω*_1_ and *ω*_2_ represent the resonant frequencies for the first (left) and second (right) resonances, respectively, while *ω*_*p*1_ and *ω*_*p*2_ are their corresponding plasma frequencies. As shown in Figure 1, the double-resonant metasurfaces with various permittivity at large dynamic region, negative region, near-zero region and positive region are behaved as series *LC* pair, inductor, parallel *LC* pair and capacitor, respectively. Specifically, in the vicinity of the resonant region of the first resonance (*ω* ≈ *ω*_1_), where the effect of the second resonance on it can be neglected, the permittivity is simplified as $\varepsilon_{m}=\varepsilon_{\infty }-\omega_{p1}^{2}/\left(\omega^{2}-\omega_{1}^{2}\right)$ , at which the permittivity has a large dynamic range (*ɛ*_*m*_ ≈ ∞) and the double-resonant metasurface behaves as a series *LC* pair with an inductance of $L_{S}=1/\omega_{p1}^{2}\varepsilon_{0}t$ and a capacitance of $C_{S}=\omega_{p1}^{2}\varepsilon_{0}t/\omega^{2}$ . With the increment of frequency (*ω*_1_ < *ω* < *ω*_0_), the relative permittivity becomes large but still remains negative values (*ɛ*_*m*_ < 0), the metasurface behaves as a nanoinductor with the inductance of $L=\left(\omega^{2}-\omega_{1}^{2}\right)/\omega^{2}\omega_{p1}^{2}\varepsilon_{0}t$ . When frequency reaches *ω*_0_, the value of the relative permittivity approaches zero, i.e., *ɛ*_*m*_ ≈ 0, the influence of the second resonance enhances and cannot be ignored, the metasurface manifests itself as a parallel *LC* pair with an inductance of $L_{P}=\left(\omega^{2}-\omega_{1}^{2}\right)/\omega^{2}\omega_{p1}^{2}\varepsilon_{0}t$ and a capacitance of $C_{P}=\omega_{p2}^{2}\varepsilon_{0}t/\left(\omega_{2}^{2}-\omega^{2}\right)$ . As the frequency continues to increase (*ω*_0_ < *ω* < *ω*_2_), the relative permittivity is with a positive value (*ɛ*_*m*_ > 0), the effect of the second resonance is dominated while that of the first resonance is neglected, thus the metasurface performs as a nanocapacitor with the capacitance of $C=\omega_{p2}^{2}\varepsilon_{0}t/\left(\omega_{2}^{2}-\omega^{2}\right)$ . The detailed theoretical derivations and analyses are demonstrated in detail in Supplementary Note 2. In doing this, complex frequency response can be easily realized by dispersion synthesizing with designed metasurfaces as lumped circuit elements.

Next, following the Butterworth filter design method [41], high-selectivity and broad out-of-band rejection filtering response can be achieved by stacking multilayer parallel *LC* pairs. Thus, it is necessary to physically implement double-resonant metasurfaces in the desired frequency regime and to establish a comprehensive table that correlates the lumped elements with the dimensions of their corresponding metasurfaces. This preliminary work facilitates the straightforward selection of the physical dimensions of corresponding metasurfaces by means of a table look-up method commonly used in electronics. This offers a considerably simple and time-efficient paradigm for high-performance metasurface design.

**Figure 2:**
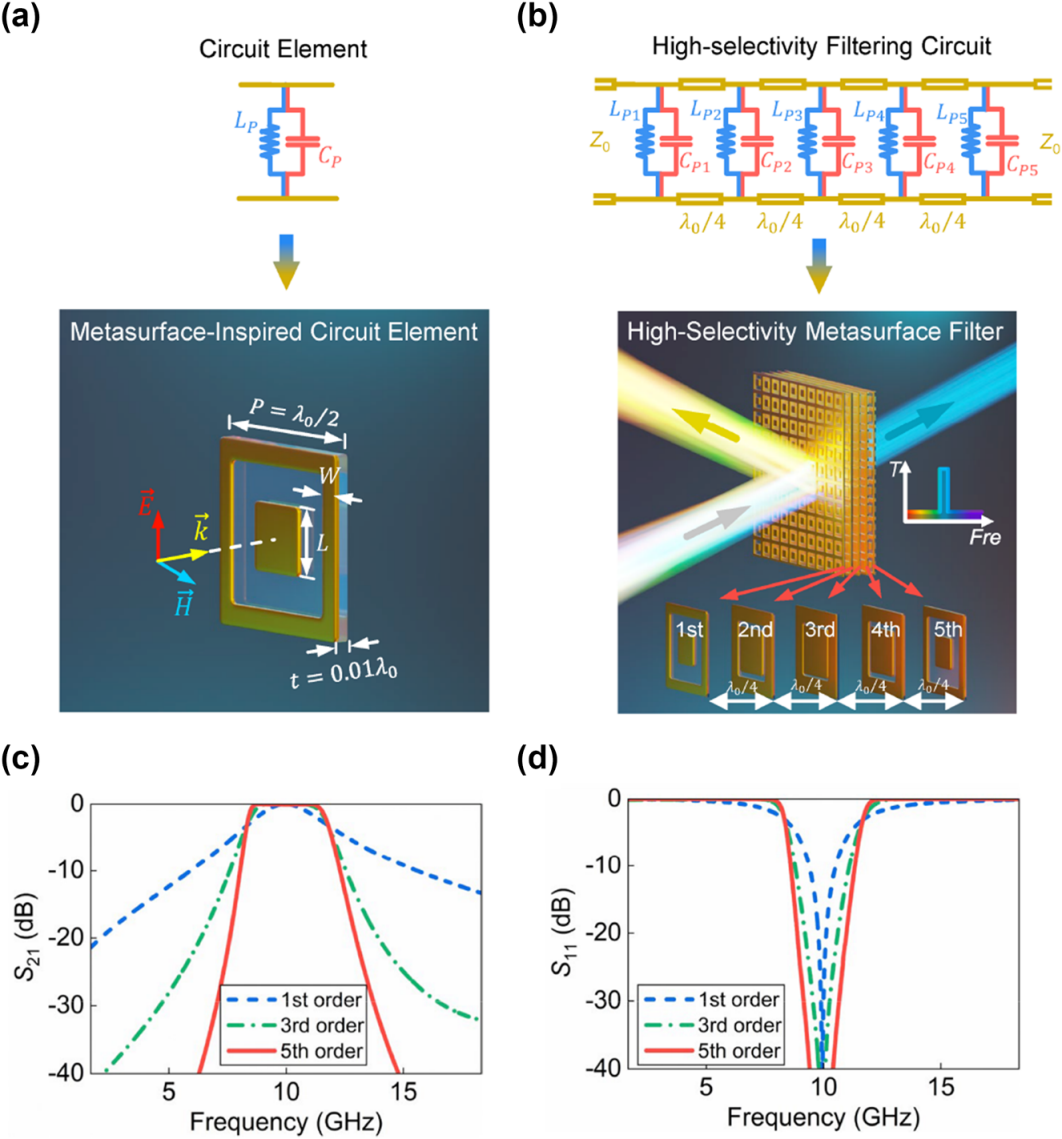
Physical implemented and filtering response of the designed high-selectivity metasurface filter. Circuit and physical implementation of the (a) metasurface-inspired circuit element and (b) high-selectivity metasurface filter. Simulated (c) transmission and (d) reflection spectra of the higher-order metasurface filters.

As shown in Figure 2(a), the physically implemented metasurfaces are designed using two metal resonators consisting of a square ring and a square patch. The first resonance is produced by the square loop, which can be regarded as an infinite-length crossed-dipole and resonates at zero frequency (*ω*_1_ = 0), while the plasma frequency (*ω*_*p*1_) is determined by the width (*W*) of the square loop. For the second resonance, its resonant and plasma frequencies (*ω*_2_ and *ω*_*p*2_) are primarily controlled by the side length (*L*) of the square patch. By adjusting the structural dimensions of employed resonators and thus tailoring the resonant parameters of the overall structures, the designed metasurfaces can behave as parallel *LC* pairs in the frequency of interest. Then, as shown in Figure 2(b), high-selectivity metasurface filter with the prescribed fractional bandwidth of 30 % and center frequency of 10 GHz is realized by stacking multi-layered metasurface-inspired circuit elements. The comprehensive table that correlates each layer of lumped elements with the dimensions of corresponding metasurfaces is provided in Supplementary Table S1.

The simulated frequency responses of the designed high-selectivity metasurface filter with high rectangular coefficients and broad out-of-band rejection properties are depicted in Figure 2(c). It can be seen that the losses of the designed filters are less than 0.5 dB for three cases. Meanwhile, the frequency-selectivity is significantly enhanced as their orders arise. Here, rectangular coefficients (*RC*) are evaluated and calculated as the ratio of the bandwidths of $\left|S_{21}\right|=-20$ dB and $\left|S_{21}\right|=-3$ dB, i.e., *RC* = *BW*_20dB_/*BW*_3dB_. Specifically, the rectangular coefficients of the designed high-selectivity metasurface filters are decreased from 5.68 to 1.49 with the increment of orders. Meanwhile, the fractional bandwidths of two sidebands are 87 % and 83 %, respectively. This results in near-rectangular-shaped filtering characteristic while simultaneously achieving broader and stronger out-of-band suppression.

## 3 Experimental results

**Figure 3:**
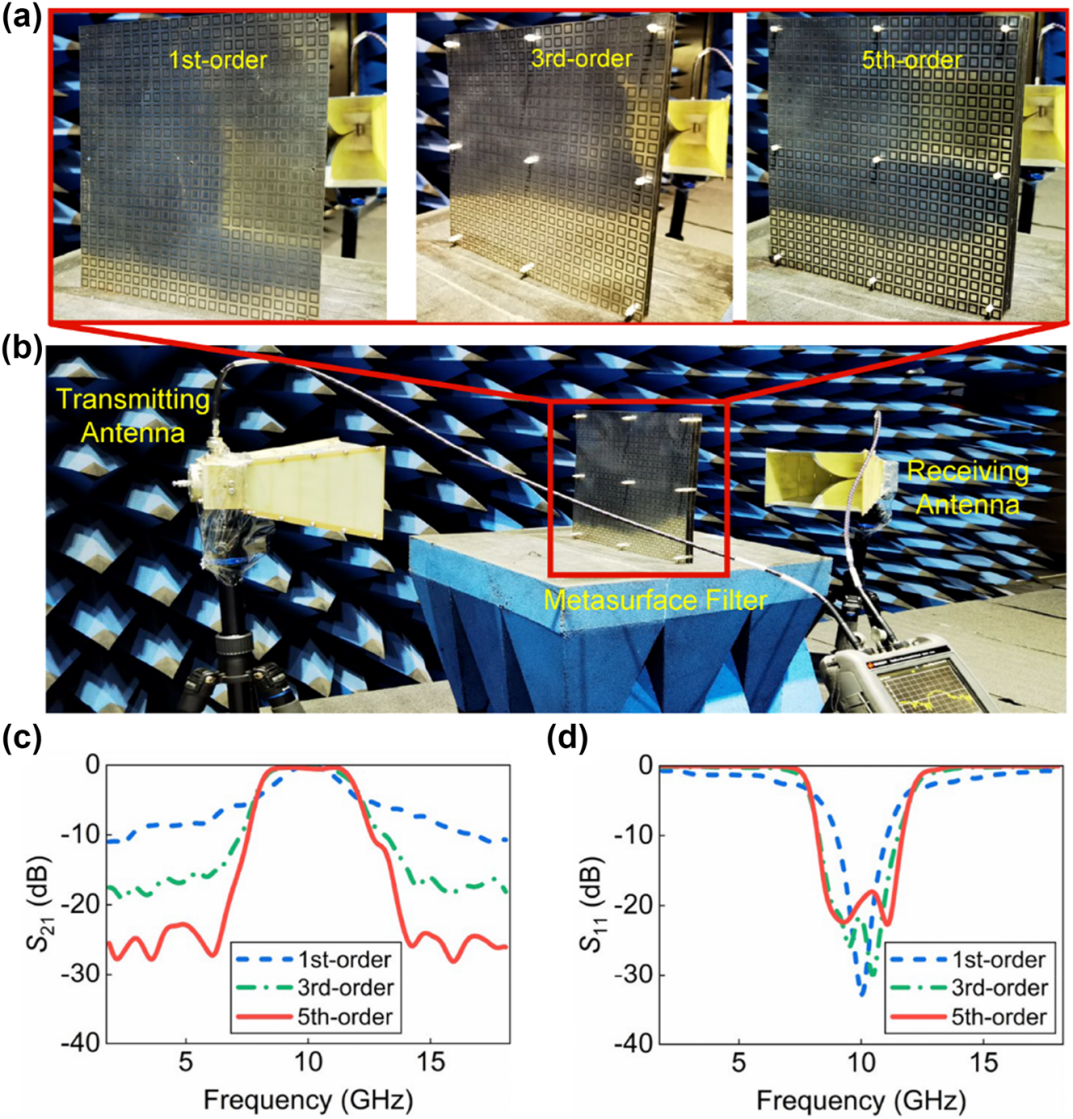
Metasurface filter fabrication, measurement setups, and experimental results. (a) Photographs of the fabricated higher-order metasurface filters for 1st-, 3rd-, and 5th-order cases. (b) Experimental setup configuration. Measured (c) transmission and (d) reflection spectra of the higher-selectivity metasurface filters.

To experimentally verify the effectiveness of the dispersion synthesis approach to achieve high-selectivity metasurface filter based on the concept of metatronics, the designed different orders of filters are fabricated and measured. As depicted in Figure 3(a), three fabricated prototypes comprise of 1-, 3- and 5-layer metasurfaces, respectively, and each layer of metasurface consists of 24 × 24 unit cells and covers an area of 300 × 300 mm^2^. In the measurement setup displayed in Figure 3(b), three samples are measured in the microwave chamber, and their measured transmittance and reflectance are obtained. In Figure 3(c) and (d), the measured results illustrate that the rectangular coefficients decrease from 5.68 to 1.49 as the filter order progresses from 1st to 5th. Concurrently, the fractional bandwidths of out-of-band suppression are quantified at 87 % and 83 % for lower and upper sidebands, respectively. In addition, the measured transmission coefficients at the two sidebands are slightly higher than the simulation results. This discrepancy is primarily ascribed to the assembly errors in the structure. The ultrathin thickness of each layer of the metasurface filters bestows a certain flexibility, leading to localized collapse during assembly due to insufficient support. This results in an uneven surface and a non-uniform distance between the adjacent layers of the designed metasurface filter, thereby influencing the out-of-band suppression.

The objective key performance indicators of the proposed high-selectivity metasurface filters, along with previously reported works, are provided in Table 1. Compared to the conventional works based on the cross-coupling method, as well as conventional optical circuits based on microrings and photonics crystals, the proposed filters exhibit higher selectivity with broader out-of-band rejection. This property facilitates the suppression of spurious signals and improves spectrum utilization efficiency, which are essential for enhancing the precision and efficiency of signal processing and providing reliable alternatives for fiber optic communications [42], [43], [44] and biomedical detectors [2]. In addition, though the out-of-band bandwidths in the proposed filter based on periodic metasurfaces are affected by the passbands located at octave positions, the out-of-band bandwidth in this work is already the theoretically widest within this limitation, without employing complex design. Meanwhile, considering the demand for flexible performance of filter circuits in practical applications, the proposed metasurface filters can be potentially generalized from integer-order to fractional-order. In specific implementation, the fractional-order frequency can be approximated as several polynomials of integer-order frequency in the frequency range of interest. In this way, the fractional-order lumped element can be realized by cascading several integer-order lumped elements, albeit at the expense of the total structural thickness.

**Table 1:** Objective key performance indicators of higher-selectivity metasurface filters together with previously works.

Literature	Operating	Method	Thickness	Rectangular	Out-of-band bandwidth		Out-of-band
	regimes		(*λ*_0_)	coefficients	Lower	Upper	attenuation
[5]	9.68–10.37 GHz	Cross-coupling	0.17	1.74	75.2 %	39 %	−16 dB
[6]	9.9–10.36 GHz	Cross-coupling	0.17	1.54	86 %	20.9 %	−13 dB
[11]	9.36–10.4 GHz	Cross-coupling	0.99	1.6	30.2 %	67.8 %	−17 dB
[22]	200.5–202 THz	Microring	10.92	1.5	4.1 %	4.5 %	−30 dB
[25]	196.5–196.8 THz	Photonics crystals	7.65	4.21	16.5 %	22.3 %	−20 dB
This work	8.6–11.6 GHz	Metatronics-inspired	1.04	1.49	87 %	83 %	−26 dB

## 4 Discussion

### 4.1 Metasurface filters in visible regime

**Figure 4:**
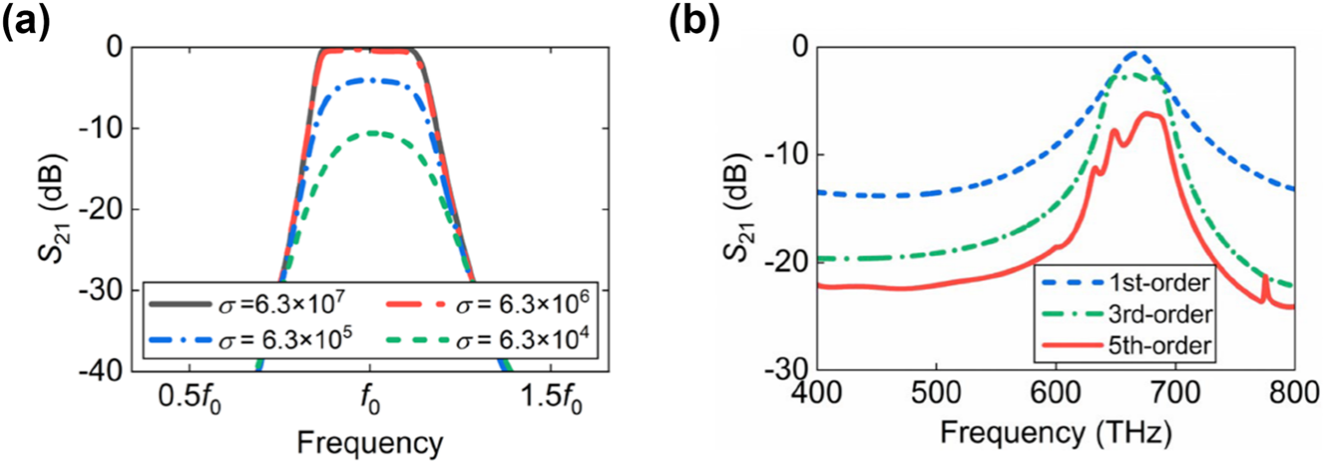
Filtering response of the designed metasurface filters at higher frequencies under the influence of varied metal conductivity. (a) The influence of the metal conductivity on the performance of the designed metatronic circuits. (b) Simulated transmittance spectra of the silver-based metasurface filters for 1st-order, 3rd-order, and 5th-order cases.

In this work, the proposed high-selectivity metasurfaces are discussed and designed only for the microwave regime. In fact, benefiting from the frequency-transferability of the metasurface, its filtering response can also be scaled to other frequency regimes such as terahertz regime, infrared regime and even up to visible regime. The difference in properties of the designed filters at different frequency regimes exists only in terms of losses due to the variation in metal conductivity circuits, as shown in Figure 4(a). In lower frequency bands, such as terahertz, millimeter, and microwave bands, the losses are with low level due to the large conductivity of the metal, which has been confirmed in the above measurements. However, at visible frequencies, metals typically suffer larger losses due to their reduced conductivity. Figure 4(b) depicts the transmission spectra of different orders of the designed metasurface filter. It is observed that the designed high-selectivity metasurface filters maintain their high selectivity and broad out-of-band suppression, despite with the increasing circuit losses. Here the metal is chosen as silver due to its significantly low losses in the visible regime, facilitating the realization of low-loss filtering properties compared to other regular metals. In addition, silver possesses very high thermal conductivity, assisting in dissipating heat from devices and reducing performance deterioration due to excessive temperatures. However, the cost and limited resources of silver may pose challenges in terms of affordability and raw material supply for its application scenarios.

### 4.2 Performance of the designed metasurface filters under oblique incidence

Angular stability is also an indicator for the proposed metasurface filters. The angular stability of the designed metasurface filter can generally be enhanced by reducing the period of the metasurface unit cell. In this work, given the structural dimensions and fabricated accuracy of the employed resonators, the period of the metasurface filter is set to 7.5 mm. Here, the angular response of the 5th-order metasurface filter is evaluated and simulated with an oblique angle range of 0–40°. As shown in Supplementary Figure S1, the in-band filtering response of the proposed metasurface filter maintains its angular stability up to 40°. However, it is noticed that the out-of-band rejection slightly degrades in high-frequency regime when the incidence angle reaches 40°. In the future, the angular stability can be further improved by employed miniaturized resonators to reduce the period of the metasurface filters.

### 4.3 Bandwidth and frequency modulation ability of the designed metasurface filters

The bandwidth of the designed metasurface filters can be modified by adjusting the slot width of the employed double-resonant metasurface. According to the Butterworth filter design method, the dimensions of the double-resonant metasurfaces are distributed in a prescribed ratio, where the slot width of the center layer is the narrowest and that of the outer layer is the widest. Therefore, the lower limit of the modulation bandwidth is determined by the narrowest achievable slot width of the center layer metasurface, which is usually limited by the fabricated accuracy and the operating frequency. The upper limit of the modulation bandwidth is determined by the widest achievable slot width of the outer layer of the metasurface, which is usually dictated by the period of the metasurface filters. In this work, considering the above limitation, the modulation bandwidth of the designed metasurface filter ranges from approximately 20 %–40 %. Supplementary Figure S2 illustrates the simulated transmission spectra of the designed metasurface filters with the fractional bandwidths of 20 % and 40 %, respectively. The circuit elements and dimensions of implemented metasurface filters are given in Supplementary Tables S2 and S3.

Similarly, by adjusting the structural dimensions, the proposed metatronic circuits with center frequencies of 8 GHz and 9 GHz are simulated, respectively. Their simulated transmittance spectra are displayed in Supplementary Figure S3, whose elements and their corresponding dimensions are given in Supporting Information Tables S4 and S5. Numerical simulations indicate that the bandwidth and center frequency of the metatronic circuits can be tailored by tuning the structural dimensions of the employed double-resonant metasurface.

### 4.4 Order limitation of the designed metasurface filters

It is worth mentioning that the high-selectivity performance achieved in this work is still not reaching its limitation, and the rectangular coefficient can be further suppressed by designing higher-order metasurface filters with more layers. However, escalating the order of the metasurface filter necessitates a corresponding increase in the slot widths of each layer of the double-resonant metasurfaces. This adjustment may mandate a larger period and thicker thickness of the metasurface filter. Given the current period of the designed metasurface filters is 7.5 mm, the maximum order achievable for the designed metasurface filter is 6th-order. Supplementary Figure S4 gives the transmission and reflection spectra of the metasurface filters for 1st-order, 3rd-order, 5th-order and 6th-order cases. It can be seen that the benefits of increasing the order of the metasurface filter on enhancing frequency selectivity become progressively marginal. Therefore, in practical design, striving for a higher order may not represent the optimal strategy when considering the holistic performance of the filter, including structural thickness and angular stability.

### 4.5 Impact of spatial dispersion in periodic metasurface

To physically implement the metatronic circuit elements, double-resonant metasurfaces with a periodic form are employed to regulate their relative permittivity, achieving the desired double-resonant dispersion. However, due to the spatial dispersion inherent in periodic resonators [45], the relative permeability fluctuates near the resonant frequency, which is inconsistent with our assumption of a nonmagnetic metasurface (*μ*_*m*_ = 1). To further investigate this, Supplementary Figure S5 plots the extracted relative permittivity and relative permeability of the employed metasurface shown in Figure 2(a). It is evident in the figure that the values of relative permeability are much smaller than those of relative permittivity, indicating that the spatial dispersion in periodic metasurface has a minimal effect on the relationship between the employed metasurface and its corresponding metatronic circuit element.

## 5 Conclusions

In summary, we have introduced an approach for dispersion synthesis with metasurfaces to achieve high-selectivity combined with broadband out-of-band rejection filtering response according to the concepts of metatronics. Based on the metatronics concept and circuit design, complex frequency response can be realized by stacking meticulously designed metasurfaces as lumped elements in electronics through controlling the displacement current flow, instead of requiring numerous numerical simulations to deal with complex many-body scattering as in conventional metasurfaces and FSSs. Meanwhile, the proposed approach can be extended to realizing other high-performance metasurfaces in the manner of circuit design. This may open up promising applications for various optical devices, including light manipulation, optoelectronic computing and storage and image processing applications.

## 6 Methods

### 6.1 Numerical simulation

Numerical simulations were performed using the commercial software high frequency structure simulator (HFSS), which employs the finite element method. The dielectric material was chosen as air to weaken its influence on the filtering response, while the metal was chosen as silver. Given that the metal losses of silver in the visible regime are higher than those in the microwave regime due to finite metal conductivity, thus in the simulations, the conductivity of silver was sourced from experimental data with the finite values rather than the infinite ones under ideal conditions in the material library of the simulation software [46].

### 6.2 Fabrication and measurement setup

For the fabrication, the design metasurface filters were fabricated using copper through the standard printed circuit board process on substrate Rogers RO5880 (*ɛ*_*r*_ = 2.2), which is only 0.127 mm thick and has negligible influence on the performance of the filtering response. The adjacent metasurface-inspired parallel *LC* pairs were then separated by Nylon pillars with 7.5 mm, which corresponds to a quarter wavelength of the center frequency (10 GHz).

For the measurement, the transmission spectra of the fabricated samples were measured in a microwave anechoic chamber using the free space method. In the experiments, the samples were placed at the center between the transmitting and receiving antennas operating at 1–18 GHz, which were connected to a vector network analyzer through 50 − Ω coaxial cables, respectively. In order to satisfy the far-field condition, the transmitting and receiving antennas are placed at the distance of 1.0 m away from the fabricated sample. To mitigate the multipath effect, the time-domain gate was activated in the Keysight N9917A vector network analyzer to smooth and obtain the transmission spectra.

## Supplementary Material

Supplementary Material Details
