# Supplementary material for: Metatronics-inspired high-selectivity metasurface filter
Source: Nanophotonics. 2024 Apr 26;13(16):2995–3003. doi: 10.1515/nanoph-2024-0123 (PMC11501889; doi:10.1515/nanoph-2024-0123)
Supplement: Supplementary file 1 — Supplementary Material Details [file j_nanoph-2024-0123_suppl_001.docx]

**Supplementary Materials for**

***Metatronics-inspired high-selectivity metasurface filter***

Qihao Lv^1^, Xu Qin^1^, Mingzhe Hu^1^, Peihang Li^1^, Yongjian Zhang^1^, and Yue Li^1,2^*

*^1^Department of Electronic Engineering, Tsinghua University, Beijing 100084, China*

*^2^Beijing National Research Center for Information Science and Technology, Beijing 100084, China.*

*lyee@tsinghua.edu.cn

**Supplementary Note 1.** Derivation of admittance of the metasurfaces.

**Supplementary Note 2.** Circuit configurations and elements of double-resonant metasurfaces.

**Supplementary Table S1.** Tabulated circuit elements and dimensions of high-order metasurface filters with a fractional bandwidth of 30% and a center frequency of 10 GHz.

**Supplementary Table S2.** Tabulated circuit elements and dimensions of high-order metasurface filters with a fractional bandwidth of 20% and a center frequency of 10 GHz.

**Supplementary Table S3.** Tabulated circuit elements and dimensions of high-order metasurface filters with a fractional bandwidth of 40% and a center frequency of 10 GHz.

Supplementary Note 1: Derivation of admittance of the metasurfaces.

The admittance of a subwavelength-scaled metastructure placed in free space is characterized by the incident electric field *E_i_* and the total reflection electric field *E_r_* and can be expressed as

$Y_{m}=Y_{0}\frac{E_{i}-E_{r}}{E_{i}+E_{r}}-Y_{0}=-Y_{0}\frac{2E_{r}}{E_{i}+E_{r}},$ (S1)

where *Y*_0_ represents the admittance of the free space, and the total reflection electric field *E_r_* is given by

$E_{r}=\frac{Z_{m}-1}{Z_{m}+1}E_{i}+\frac{4Z_{m}\left(1-Z_{m}\right)}{\left(Z_{m}+1\right)^{3}}\frac{e^{j2k_{m}t}}{1-\left(\frac{1-Z_{m}}{1+Z_{m}}\right)^{2}e^{j2k_{m}t}}E_{i},$ (S2)

where $t$ denotes the metastructure thickness, *Z*_m_=√(*μ*_m_/*ε*_m_) and *k*_m_=2π√(*μ*_m_*ε*_m_)/*λ* represent the impedance and wave number in the metastructure, respectively, while *ε*_m_ and *μ*_m_ signify the relative permittivity and permeability of the metastructure, respectively. Then the admittance *Y*_m_ is simplified and written as

$Y_{m}=-Y_{0}\frac{2E_{r}}{E_{i}+E_{r}}=-Y_{0}\frac{\left(1-Z_{m}^{2}\right)j\sin k_{m}t}{Z_{m}\left(\cos k_{m}t-jZ_{m}\sin k_{m}t\right)}.$ (S3)

For a subwavelength-scaled metastructure, i.e., *k*_m_*t*≪1, the denominator of Equation S3 degenerates to *Z*_m_cos*k*_m_*t* when the metastructure satisfies the condition of *μ*_m_*t*≪*λ*/2π*t*, which is readily satisfied in nonmagnetic metastructures with subwavelength scale. Thus, the admittance of metastructure can be derived as

$$Y_{m}=-Y_{0}\frac{\left(1-Z_{m}^{2}\right)j\sin k_{m}t}{Z_{m}\cos k_{m}t}=-\sqrt{\frac{\varepsilon_{0}}{\mu_{0}}}\frac{\left(1-\frac{\mu_{m}}{\varepsilon_{m}}\right)}{\sqrt{\frac{\mu_{m}}{\varepsilon_{m}}}}\cdot jk_{m}t=-\sqrt{\frac{\varepsilon_{0}}{\mu_{0}}}\frac{\left(1-\frac{\mu_{m}}{\varepsilon_{m}}\right)}{\sqrt{\frac{\mu_{m}}{\varepsilon_{m}}}}\cdot j\frac{2\pi\sqrt{\varepsilon_{m}\mu_{m}}f}{c}t$$

$=-j\omega t\sqrt{\frac{\varepsilon_{0}}{\mu_{0}}}\left(\sqrt{\frac{\varepsilon_{m}}{\mu_{m}}}-\sqrt{\frac{\mu_{m}}{\varepsilon_{m}}}\right)\cdot\sqrt{\varepsilon_{m}\mu_{m}}\cdot\sqrt{\varepsilon_{0}\mu_{0}}$ (S4)

Then, we have

$Y_{m}=-j\omega\left(\varepsilon_{m}-\mu_{m}\right)\varepsilon_{0}t.$ (S5)

According to the equations above, the circuit properties of the subwavelength-scaled metastructures are characterized with the aids of their relative permittivity and permeability.

Supplementary Note 2: Circuit configurations and elements of double-resonant metasurfaces.

For a subwavelength-scaled nonmagnetic metastructure (*μ*_m_ = 1) with double-resonant Drude-Lorentz dispersion, its relative permittivity is written as

$\varepsilon_{m}=\varepsilon_{\infty}-\frac{\omega_{p1}^{2}}{\omega^{2}-\omega_{1}^{2}}-\frac{\omega_{p2}^{2}}{\omega^{2}-\omega_{2}^{2}}\text{，}$ (S6)

where *ε*_∞_ = 1 is the relative permittivity at infinite frequency, *ω*_1_ and *ω*_2_ represent the resonant frequencies for the first (left) and second (right) resonances, respectively, while *ω*_p1_ and *ω*_p2_ are their corresponding plasma frequencies.

In the vicinity of resonant region of the first resonance (*ω* ≈ *ω*_1_), where the effect of the second resonance on it can be neglected, the permittivity is simplified as *ε*_m_=*ε*_∞_-*ω*_p1_^2^/(*ω*^2^-*ω*_1_^2^). Then, the admittance of the Drude-Lorentz metastructure can be obtained as:

$$Y_{m}=-j\omega\left(\varepsilon_{m}-\mu_{m}\right)\varepsilon_{0}t=-j\omega\left(\varepsilon_{\infty}-\frac{\omega_{p1}^{2}}{\omega^{2}-\omega_{1}^{2}}-\mu_{m}\right)\varepsilon_{0}t=j\omega\frac{\omega_{p1}^{2}}{\omega^{2}-\omega_{1}^{2}}\varepsilon_{0}t$$

$=\frac{1}{-j\omega\frac{1}{\omega_{p1}^{2}\varepsilon_{0}t}-\frac{1}{j\omega\frac{\omega_{p1}^{2}\varepsilon_{0}t}{\omega^{2}}}}=\frac{1}{-j\omega L_{S}-\frac{1}{j\omega C_{S}}}.$ (S7)

As demonstrated in equation (S7), the metastructure behaves as a metatronic LC pair with the series configuration near the resonant region. The capacitance *C*_S_ and inductance *L*_S_ of the proposed single-resonant metastructure are expressed as

$L_{S}=1/\omega_{p1}^{2}\varepsilon_{0}t \text{and} C_{S}=\omega_{p1}^{2}\varepsilon_{0}t/\omega^{2}.$ (S8)

When the metastructure operate at higher frequencies (*ω*_1_<*ω*<*ω*_0_), the relative permittivity becomes large but remains negative values (*ε*_m_*<*0). In this case, the admittance of the Drude-Lorentz metastructure can be rewritten as:

$Y_{m}=-j\omega\left(\varepsilon_{m}-\mu_{m}\right)\varepsilon_{0}t=j\omega\frac{\omega_{p1}^{2}}{\omega^{2}-\omega_{1}^{2}}\varepsilon_{0}t=\frac{1}{-j\omega\frac{\omega^{2}-\omega_{1}^{2}}{\omega^{2}\omega_{p1}^{2}\varepsilon_{0}t}}=\frac{1}{-j\omega L}.$ (S9)

In this frequency region, the metastructure behaves as a nanoinductor, whose inductance is calculated as

$L=\left(\omega^{2}-\omega_{1}^{2}\right)/\omega^{2}\omega_{p1}^{2}\varepsilon_{0}t.$ (S10)

When the operating frequency reach the frequency *ω*_0_, the value of the relative permittivity approaches zero, i.e., *ε_m_* ≈ 0, the influence of the second resonance enhances and cannot be ignored, the admittance of the Drude-Lorentz metastructure is expressed as:

$$Y_{m}=-j\omega\left(\varepsilon_{m}-\mu_{m}\right)\varepsilon_{0}t=j\omega\left(\frac{\omega_{p1}^{2}}{\omega^{2}-\omega_{1}^{2}}+\frac{\omega_{p2}^{2}}{\omega^{2}-\omega_{2}^{2}}\right)\varepsilon_{0}t$$

$=-\frac{1}{j\omega\frac{\omega^{2}-\omega_{1}^{2}}{\omega^{2}\omega_{p1}^{2}\varepsilon_{0}t}}-j\omega\frac{\omega_{p2}^{2}\varepsilon_{0}t}{\omega_{2}^{2}-\omega^{2}}=-\frac{1}{j\omega L_{P}}-j\omega C_{P}.$ (S11)

Equation (S11) demonstrates that the Drude-Lorentz metastructure performs as a metatronic LC pair with the parallel configuration near the frequency *ω*_0_. Thus, the capacitance *C*_P_ and inductance *L*_P_ of the proposed double-resonant metastructure are expressed as

$L_{P}=\left(\omega^{2}-\omega_{1}^{2}\right)/\omega^{2}\omega_{p1}^{2}\varepsilon_{0}t \text{and} C_{P}=\omega_{p2}^{2}\varepsilon_{0}t/\left(\omega_{2}^{2}-\omega^{2}\right).$ (S12)

As the operating frequency continues to increase (*ω*_0_<*ω*<*ω*_2_), the relative permittivity is with a positive value, the effect of the second resonance is dominated while that of the first resonance is neglected. Thus, the relative permittivity of the metastructure can be simplified in another form of*ε*_m_=*ε*_∞_-*ω*_p2_^2^/(*ω*^2^-*ω*_2_^2^), and its admittance is rewritten as

$Y_{m}=-j\omega\left(\varepsilon_{m}-\mu_{m}\right)\varepsilon_{0}t=j\omega\left(\frac{\omega_{p2}^{2}}{\omega^{2}-\omega_{2}^{2}}\right)\varepsilon_{0}t=-j\omega C$ (S13)

In this frequency regime, the Drude-Lorentz metastructure behaves as a parallel capacitor with the capacitance of

$C=\omega_{p2}^{2}\varepsilon_{0}t/\left(\omega_{2}^{2}-\omega^{2}\right).$ (S14)

**
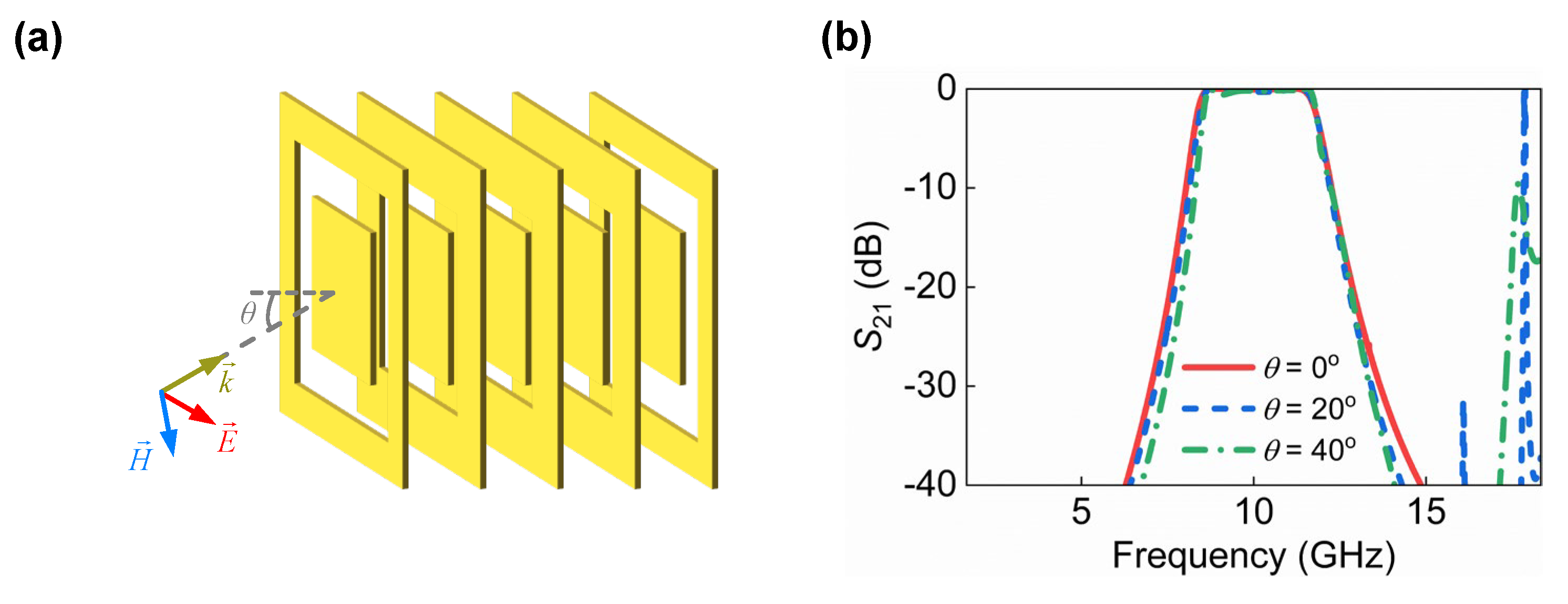
**

**Figure S1:** Filtering response of different orders of the designed metasurface filters under oblique incidence. a) Schematic diagram of the proposed 5th-order metasurface filter under oblique incidence. b) Transmittance spectra of the proposed 5th-order metasurface filter with a range of incidence angle of 0-40^o^.


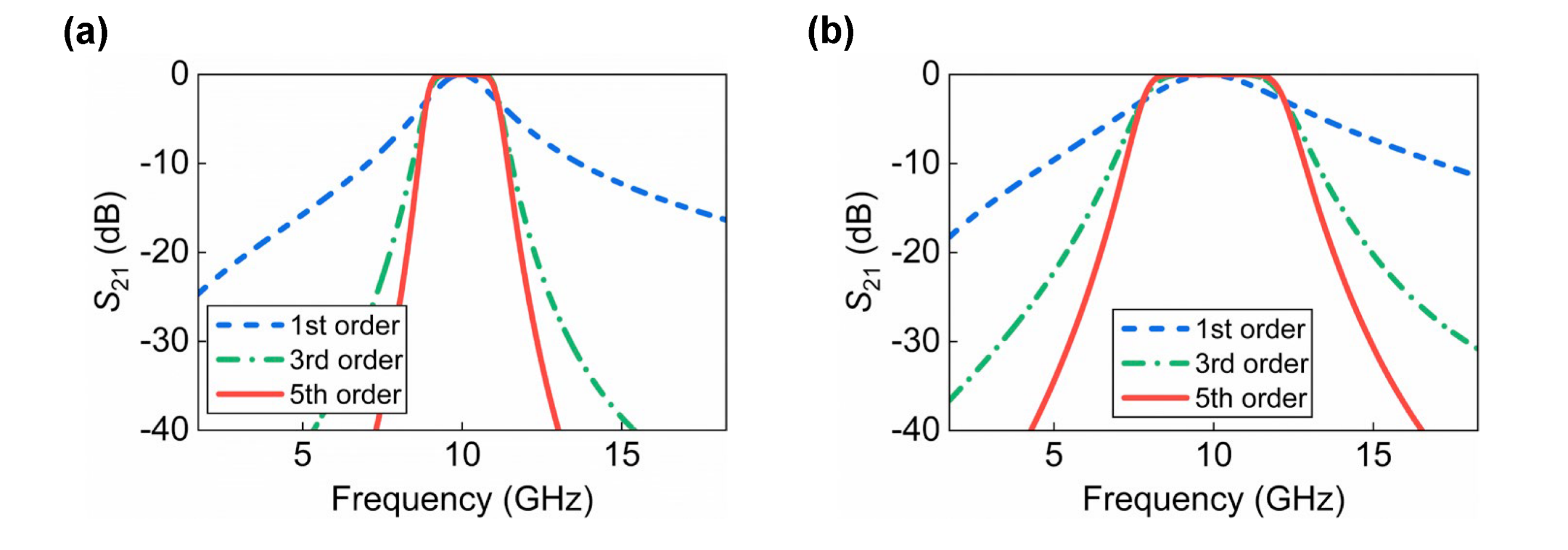


**Figure S2:** Filtering response of different orders of the designed metasurface filters with diverse fractional bandwidths of a) 20% and b) 40%.

**
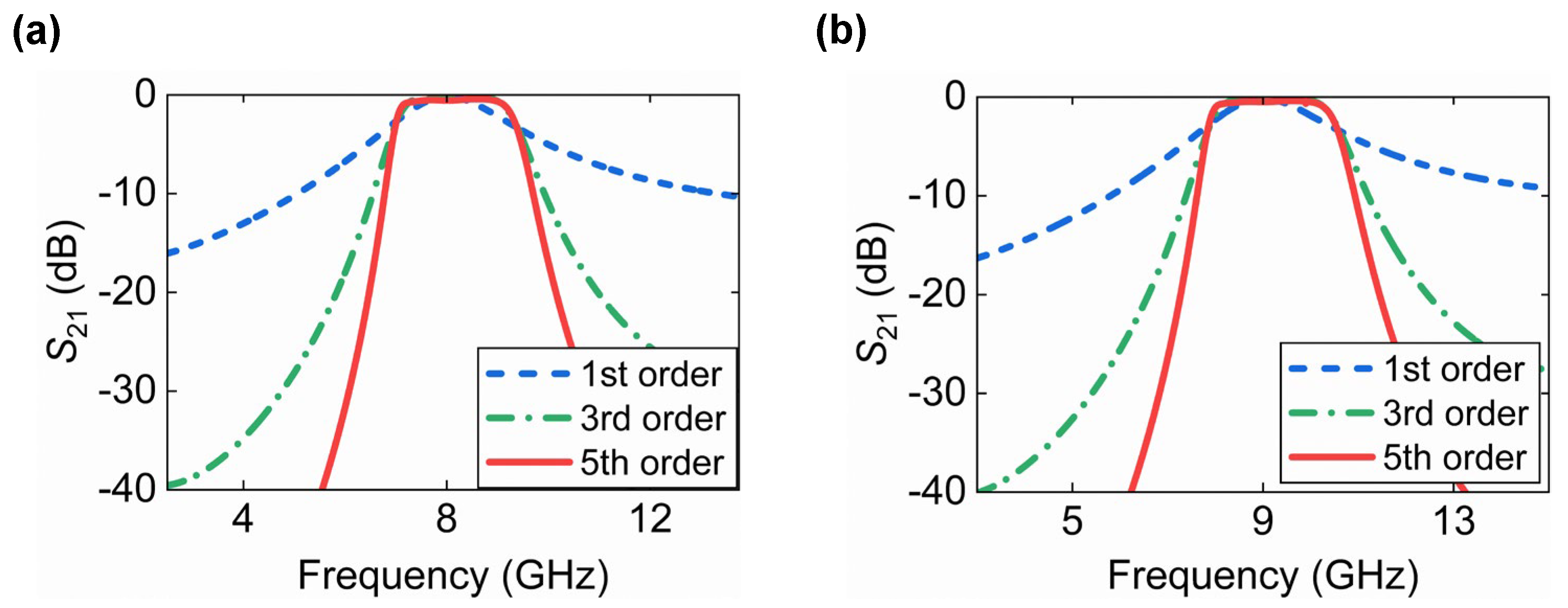
**

**Figure S3:** Filtering response of different orders of the designed metasurface filters with diverse center frequencies of a) 8 GHz and b) 9 GHz.

**
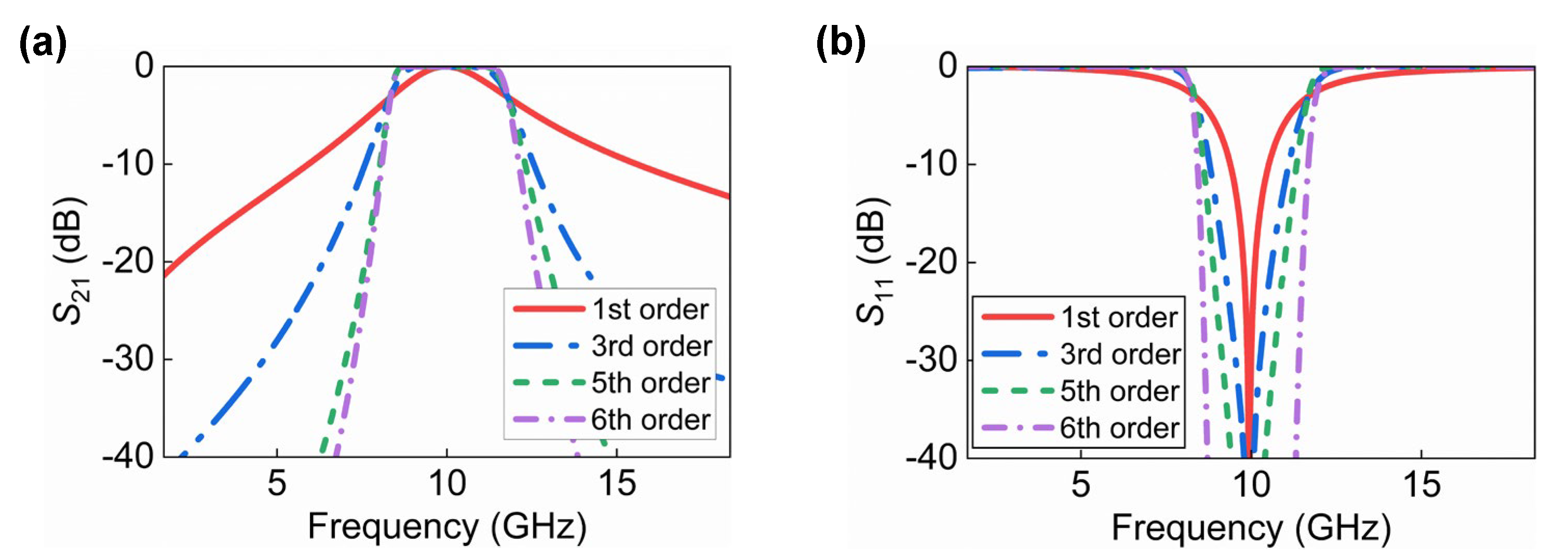
**

**Figure S4:** Transmission and reflection spectra of the proposed metasurface for 1st-order, 3rd-order, 5th-order, and 6th-order cases. a) Transmission and b) reflection spectra.


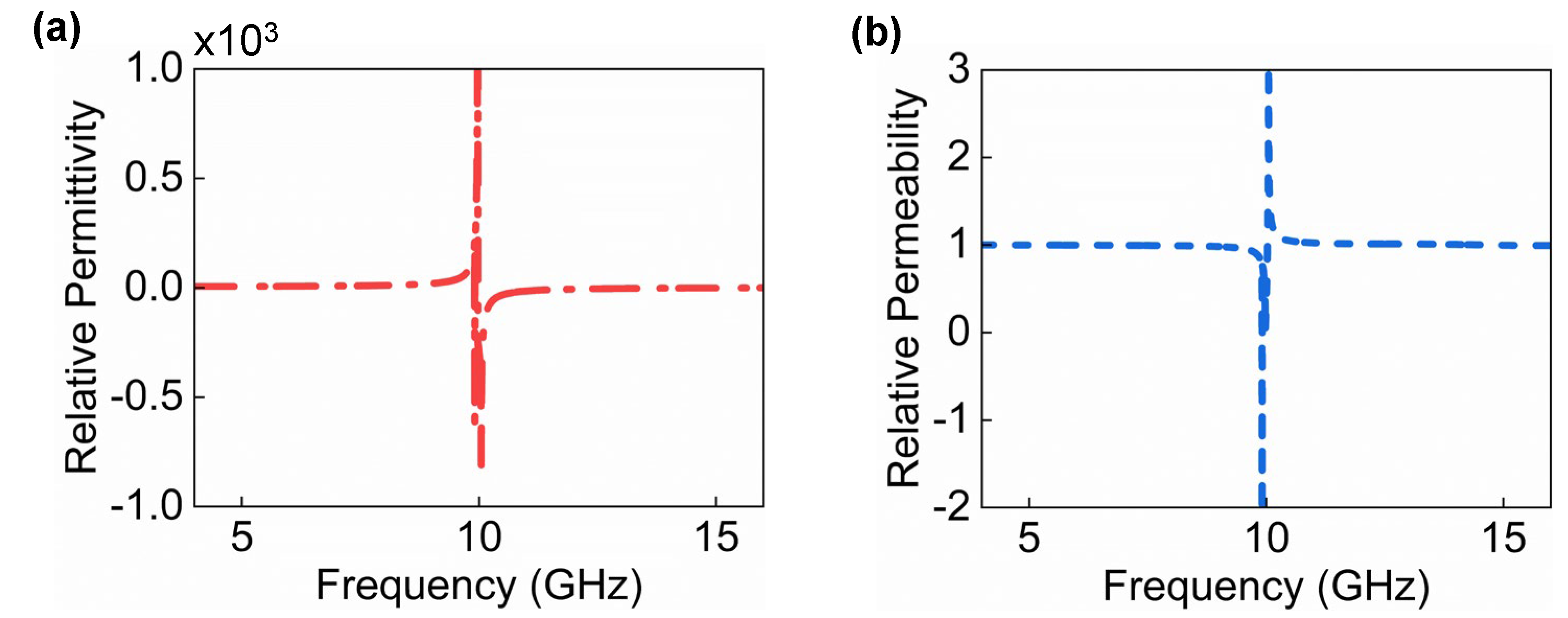


Figure S5: The relative permittivity and permeability of the employed double-resonant metasurface under the effect of spatial dispersion. a) Relative permittivity. b) Relative permeability.

Table S1 Tabulated circuit elements and dimensions of high-order metasurface filters with a fractional bandwidth of 30% and a center frequency of 10 GHz.

| Filter  order | Layer No. | *L_P_*/*λ*_0_  (nH·m^-1^) | *C_P_*/*λ*_0_  (pF·m^-1^) | *L*/*λ*_0_ | *W*/*λ*_0_ |
| --- | --- | --- | --- | --- | --- |
| 1st | Layer 1 | 30.0 | 9.38 | 0.24 | 0.06 |
| 3rd | Layer 1, 3 | 60.0 | 4.69 | 0.27 | 0.1 |
|  | Layer 2 | 30.0 | 9.38 | 0.24 | 0.06 |
| 5th | Layer 1, 5 | 97.1 | 2.90 | 0.29 | 0.12 |
|  | Layer 2, 4 | 37.1 | 7.59 | 0.26 | 0.07 |
|  | Layer 3 | 30.0 | 9.38 | 0.24 | 0.06 |

Table S2 Tabulated circuit elements and dimensions of high-order metasurface filters with a fractional bandwidth of 20% and a center frequency of 10 GHz.

| Filter  order | Layer No. | *L_P_*/*λ*_0_  (nH·m^-1^) | *C_P_*/*λ*_0_  (pF·m^-1^) | *L*/*λ*_0_ | *W*/*λ*_0_ |
| --- | --- | --- | --- | --- | --- |
| 1st | Layer 1 | 20.0 | 14.07 | 0.22 | 0.03 |
| 3rd | Layer 1, 3 | 40.0 | 7.04 | 0.25 | 0.05 |
|  | Layer 2 | 20.0 | 14.07 | 0.22 | 0.03 |
| 5th | Layer 1, 5 | 64.73 | 4.35 | 0.27 | 0.06 |
|  | Layer 2, 4 | 24.72 | 11.38 | 0.24 | 0.04 |
|  | Layer 3 | 20.0 | 14.07 | 0.22 | 0.03 |

Table S3 Tabulated circuit elements and dimensions of high-order metasurface filters with a fractional bandwidth of 40% and a center frequency of 10 GHz.

| Filter  order | Layer No. | *L_P_*/*λ*_0_  (nH·m^-1^) | *C_P_*/*λ*_0_  (pF·m^-1^) | *L*/*λ*_0_ | *W*/*λ*_0_ |
| --- | --- | --- | --- | --- | --- |
| 1st | Layer 1 | 40.0 | 7.04 | 0.27 | 0.08 |
| 3rd | Layer 1, 3 | 80.0 | 3.52 | 0.3 | 0.12 |
|  | Layer 2 | 40.0 | 7.04 | 0.27 | 0.08 |
| 5th | Layer 1, 5 | 129.45 | 2.17 | 0.32 | 0.15 |
|  | Layer 2, 4 | 49.44 | 5.69 | 0.29 | 0.1 |
|  | Layer 3 | 40.0 | 7.04 | 0.27 | 0.08 |

Table S4 Tabulated circuit elements and dimensions of high-order metasurface filters with a fractional bandwidth of 30% and a center frequency of 8 GHz.

| Filter  order | Layer No. | *L_P_*/*λ*_0_  (nH·m^-1^) | *C_P_*/*λ*_0_  (pF·m^-1^) | *l_p_*/*λ*_0_ | *w_p_*/*λ*_0_ |
| --- | --- | --- | --- | --- | --- |
| 1st | Layer 1 | 30.0 | 9.38 | 0.25 | 0.06 |
| 3rd | Layer 1, 3 | 60.0 | 4.69 | 0.28 | 0.1 |
|  | Layer 2 | 30.0 | 9.38 | 0.25 | 0.06 |
| 5th | Layer 1, 5 | 97.1 | 2.90 | 0.3 | 0.12 |
|  | Layer 2, 4 | 37.1 | 7.59 | 0.26 | 0.07 |
|  | Layer 3 | 30.0 | 9.38 | 0.25 | 0.06 |

Table S5 Tabulated circuit elements and dimensions of high-order metasurface filters with a fractional bandwidth of 30% and a center frequency of 9 GHz.

| Filter  order | Layer No. | *L_P_*/*λ*_0_  (nH·m^-1^) | *C_P_*/*λ*_0_  (pF·m^-1^) | *l_p_*/*λ*_0_ | *w_p_*/*λ*_0_ |
| --- | --- | --- | --- | --- | --- |
| 1st | Layer 1 | 30.0 | 9.38 | 0.25 | 0.07 |
| 3rd | Layer 1, 3 | 60.0 | 4.69 | 0.28 | 0.1 |
|  | Layer 2 | 30.0 | 9.38 | 0.25 | 0.07 |
| 5th | Layer 1, 5 | 97.1 | 2.90 | 0.3 | 0.12 |
|  | Layer 2, 4 | 37.1 | 7.59 | 0.26 | 0.07 |
|  | Layer 3 | 30.0 | 9.38 | 0.25 | 0.07 |
